# Cu(ATSM) Increases P-Glycoprotein Expression and Function at the Blood-Brain Barrier in C57BL6/J Mice

**DOI:** 10.3390/pharmaceutics15082084

**Published:** 2023-08-03

**Authors:** Jae Pyun, HuiJing Koay, Pranav Runwal, Celeste Mawal, Ashley I. Bush, Yijun Pan, Paul S. Donnelly, Jennifer L. Short, Joseph A. Nicolazzo

**Affiliations:** 1 Drug Delivery, Disposition and Dynamics, Monash Institute of Pharmaceutical Sciences, Monash University, Parkville, VIC 3052, Australia; jae.pyun@monash.edu (J.P.); pranav.runwal1@monash.edu (P.R.);; 2 Bio21 Molecular Science and Biotechnology Institute, University of Melbourne, Parkville, VIC 3052, Australiapauld@unimelb.edu.au (P.S.D.); 3 Oxidation Biology Lab, Melbourne Dementia Research Centre, Florey Institute of Neuroscience and Mental Health, University of Melbourne, Parkville, VIC 3052, Australia; celeste.mawal@florey.edu.au (C.M.); ashley.bush@florey.edu.au (A.I.B.); 4 Drug Discovery Biology, Monash Institute of Pharmaceutical Sciences, Monash University, Parkville, VIC 3052, Australia; jennifer.short@monash.edu

**Keywords:** blood-brain barrier, P-glycoprotein, copper, bis(thiosemicarbazone), CNS drug delivery

## Abstract

P-glycoprotein (P-gp), expressed at the blood-brain barrier (BBB), is critical in preventing brain access to substrate drugs and effluxing amyloid beta (Aβ), a contributor to Alzheimer’s disease (AD). Strategies to regulate P-gp expression therefore may impact central nervous system (CNS) drug delivery and brain Aβ levels. As we have demonstrated that the copper complex copper diacetyl bis(4-methyl-3-thiosemicarbazone) (Cu(ATSM)) increases P-gp expression and function in human brain endothelial cells, the present study assessed the impact of Cu(ATSM) on expression and function of P-gp in mouse brain endothelial cells (mBECs) and capillaries in vivo, as well as in peripheral organs. Isolated mBECs treated with Cu(ATSM) (100 nM for 24 h) exhibited a 1.6-fold increase in P-gp expression and a 20% reduction in accumulation of the P-gp substrate rhodamine 123. Oral administration of Cu(ATSM) (30 mg/kg/day) for 28 days led to a 1.5 & 1.3-fold increase in brain microvascular and hepatic expression of P-gp, respectively, and a 20% reduction in BBB transport of [3H]-digoxin. A metallomic analysis showed a 3.5 and 19.9-fold increase in Cu levels in brain microvessels and livers of Cu(ATSM)-treated mice. Our findings demonstrate that Cu(ATSM) increases P-gp expression and function at the BBB in vivo, with implications for CNS drug delivery and clearance of Aβ in AD.

## 1. Introduction

The blood-brain barrier (BBB) consists of specialised endothelial cells lining the network of microvessels that perfuse the brain and has an essential role in regulating the interface between the central nervous system (CNS) and the systemic circulation [1]. Brain microvascular endothelial cells express various efflux transporter proteins on their luminal membrane, such as P-glycoprotein (P-gp), which acts as a “gatekeeper” to prevent the CNS entry of a wide range of xenobiotic molecules [2]. P-gp is the most well characterised of the ATP-binding cassette transporter family, encoded by the MDR1 gene and is also expressed in other tissues including the liver, kidneys, small intestines, placenta, and blood-testis barrier [3,4]. The brain uptake of structurally diverse compounds (dexamethasone, digoxin, cyclosporin A, ondansetron, and loperamide) has been shown to increase between twenty to one hundred-fold more in P-gp deficient mice compared with wild-type mice [4,5,6,7]. Thus, whilst P-gp has a protective role in keeping toxic substrates from entering the CNS, it also poses a major challenge for the delivery of therapeutic agents to their corresponding target sites.

Efforts to inhibit the P-gp-mediated efflux of drugs have been trialled as a means of enhancing CNS therapeutic access, but these have failed to improve clinical outcomes [8,9,10]. Therefore, to overcome the impact of P-gp on drug delivery to the brain, novel strategies to regulate P-gp expression and function are needed, in conjunction with developing new techniques to overcome the restrictive nature of the BBB, such as the use of carrier molecules, focused-ultrasound and nanoparticles [11,12,13]. However, on the other hand, reduced function or expression of P-gp can result in significant neurotoxicity and undesirable pharmacodynamic effects, particularly given that this key efflux transporter is known to be disrupted in certain disease states, leading to changes in the exchange of molecules between the blood and brain. 

There is compelling preclinical and clinical evidence to indicate that dysregulation of P-gp at the BBB contributes to the pathogenesis of neurodegenerative diseases, including Alzheimer’s disease (AD) [14,15,16,17]. Research suggests that the BBB may be disrupted in the earliest stages of AD, even before the onset of cognitive symptoms [18,19]. This early BBB disruption may contribute to the accumulation of amyloid beta (Aβ), which is a hallmark neurotoxic peptide that accumulates in the brain of individuals with AD and leads to disease progression [20,21]. P-gp plays a crucial role in the efflux of Aβ peptides and has been shown in vitro to directly transport the toxic 40 and 42 amino acid isoforms of Aβ (Aβ1-40 and Aβ1-42) [22,23]. Further studies have confirmed that Aβ is indeed a substrate of P-gp and that P-gp-mediated clearance of fluorescent Aβ can be reduced in isolated mouse brain microvessels in the presence of P-gp inhibitors [24,25]. One study demonstrated elevated endogenous Aβ levels in the hippocampal area of P-gp deficient mice indicating reduced clearance, while concurrently, administration of P-gp inhibitors also significantly increased endogenous brain levels in a mouse model of AD, clearly indicating the role of P-gp in the clearance of Aβ [26]. Clinically, there is evidence of an inverse correlation between P-gp expression and brain Aβ deposition in individuals with AD, where decreased P-gp abundance in brain microvessel regions was associated with increased Aβ plaque burden, a finding observed in numerous independent studies comparing AD and non-AD post mortem brains [27,28,29,30]. In addition to reduced P-gp expression, the efflux function of P-gp appears diminished in individuals with AD, with positron emission tomography imaging demonstrating an increased brain accumulation of the P-gp substrate [^11^C]verapamil compared to those without AD [17]. Therefore, given that P-gp mediates Aβ efflux, and P-gp expression and function are reduced in AD, increasing P-gp expression and function could be a promising approach to increase Aβ clearance and attenuate amyloid burden in individuals with AD. Targeting intracellular signalling pathways known to upregulate and restore P-gp function led to a reduction in Aβ accumulation in an AD mouse model, suggesting P-gp as a potential therapeutic target in AD [24]. Therefore, investigating approaches to restore P-gp expression and function, which could modulate the access of drugs into the CNS but also enhance the brain clearance of Aβ in AD, is warranted.

To this end, our laboratory has had an ongoing interest in the role of copper (Cu) in transporter regulation. Cu is an essential biometal that maintains brain health and neuronal integrity and modulates various regulatory pathways within the CNS [31,32]. The biodistribution of Cu is disrupted in AD [33,34], and more recently, Cu levels have been proposed to have a protective effect against cognitive decline in AD clinically [35]. Despite this, the effects of Cu and Cu-complexes on brain endothelial cells, particularly given its need to cross the BBB to reach targets in the CNS, are not well understood. Our laboratory previously demonstrated an association between intracellular levels of Cu and P-gp expression and function in a human brain microvascular endothelial (hCMEC/D3) cell line, a model representing the BBB. A metallochaperone clioquinol was utilised in combination with Cu and zinc (Zn), two essential biometals important in maintaining the health of the CNS, where increased Cu levels were associated with increased P-gp expression [36]. To further ascertain this relationship, we assessed the impact of copper diacetyl bis (4-methyl-3-thiosemicarbazone) (Cu(ATSM)) on P-gp expression and function, as this bis (thiosemicarbazone) complex has been shown to deliver exogenously bound Cu, increasing the levels of the biometal in brain cells [37]. Cu(ATSM) is a low molecular weight, highly lipophilic, charge neutral compound that crosses the BBB [38,39]. We have reported the effects of Cu(ATSM) on hCMEC/D3 cells, where we showed a 6-fold increase in Cu in hCMEC/D3 cells, and this was associated with a 2-fold increase in expression and 1.3-fold increase in function of P-gp [40]. However, as described in the previous study, this increase was not simply a result of increasing intracellular Cu as other compounds known to increase Cu did not upregulate P-gp. The combination of the Cu and the backbone structure of the ATSM complex increased P-gp expression and function and this was shown to be mediated partly through activation (1.4-fold) of the extracellular signal-regulated kinase 1 and 2 (ERK1/2) [40]. Given the previous studies were undertaken in human brain endothelial cells, the aim of the present study was to examine the effects of Cu(ATSM) on P-gp expression and function in an in vivo mouse model. This study is significant because multiple studies have demonstrated discrepancies and a lack of translation between human and rodent systems [41,42]. These studies are the first to assess the effect of Cu(ATSM) at the mouse BBB and the findings have the potential to validate a novel therapeutic approach to restore the function of a key efflux transporter, which may result in enhanced clearance of Aβ from the brain.

## 2. Materials and Methods

### 2.1. Materials

Ammonium persulfate (APS), beta-mercaptoethanol (β-ME), dimethyl sulfoxide (DMSO), Dulbecco’s phosphate buffer saline (D-PBS), glycerol, glycine, 4% (*w*/*v*) paraformaldehyde (PFA), cOmplete™ mini protease inhibitor, phosSTOP™ phosphatase inhibitor cocktail tablets, rhodamine 123 (R123), sodium chloride, sodium dodecyl sulphate (SDS), tetramethylethylenediamine (TEMED), thiazolyl blue tetrazolium bromide (MTT), tris base (TRIZMA^®^ base), Triton^®^ X-100, Tween^®^ 20, and trypan blue were purchased from Sigma-Aldrich (St Louis, MO, USA). GFAP antibody (RRID: AB_1074620), HuC/D pan-neuronal antibody (RRID: AB_221448), Iba1 antibody (RRID: AB_11156585), 0.25% trypsin-EDTA (*w*/*v*), Mem-PER™ Plus Membrane Protein Extraction kit, Pierce™ bicinchoninic acid (BCA) protein assay kit, and Pierce™ IP lysis buffer composed of 1 mM EDTA, 150 mM NaCl, 1% (*w*/*v*) NP-40 and 25 mM Tris-HCl pH 7.4 were purchased from Thermo Fisher Scientific (Rockford, IL, USA). HyClone bovine serum albumin (BSA) was purchased from GE Healthcare Life Sciences (Little Chalfont, UK). Extra thick blot paper, Mini-Protein^®^ TGX™ Precast gels (4–15% acrylamide), 0.45 µm nitrocellulose membranes, and Precision Plus Protein™ Dual Xtra Standards were purchased from Bio-Rad (Hercules, CA, USA). Primary C219 monoclonal antibody to P-gp (RRID: AB_2565033) and purified anti-mouse CD31 antibody (RRID: AB_312908) were purchased from BioLegend^®^ (San Diego, CA, USA), and the primary mouse antibody for β-actin (RRID: AB_303668) and the sodium-potassium ATPase antibody (ab76020) were purchased from Abcam (Cambridge, UK). Intercept^®^ blocking buffer in PBS, IRDye^®^ 800CW goat anti-mouse (RRID: AB_621842), and IRDye^®^ 680LT donkey anti-rabbit (RRID: AB_10706167) secondary antibodies were purchased from LI-COR (Lincoln, NE, USA). Endothelial basal medium-2 (EBM2) media was used for all primary mouse brain endothelial cell (mBEC) culture experiments, and this growth media was supplemented with 1% (*v*/*v*) penicillin/streptomycin and 10 mM HEPES purchased from Sigma-Aldrich (St Louis, MO, USA), and EGM™-2 SingleQuot™ Kit (ascorbic acid, b-slice variant fibroblast growth factor, epidermal growth factor, fetal bovine serum (FBS), gentamicin/amphotericin, hydrocortisone, insulin-like growth factor, vascular endothelial growth factor) purchased from Lonza (Walkersville, MD, USA). T75 flasks, 6 and 96-well plates, rat-tail collagen Type I and all plastic cell culture equipment were purchased from Corning (Corning, NY, USA). Dulbecco’s Modified Eagle’s Medium (DMEM) GlutaMAX™ and Hanks balanced salt solution (HBSS) were purchased from Life Technologies (Carlsbad, CA, USA). Adult Brain Dissociation kit, MACS^®^ CD31+ MicroBeads, PE CD31+ fluorophore for mouse and all MACS^®^ equipment for the magnetic-activated cell sorting for the isolation of mBECs was purchased from Miltenyi Biotech (Bergish Gladbach, Germany). [3H]-digoxin and [14C]-sucrose were purchased from American Radiolabelled Chemicals (St Louis, MO, USA) and Ultima Gold™ liquid scintillation cocktail was purchased from Perkin-Elmer Life Sciences (Waltham, MA, USA). Copper diacetyl bis(4-methyl-3-thiosemicarbazone) (Cu(ATSM)) was synthesised as previously described [43,44].

### 2.2. Isolation and Characterisation of mBECs

All experiments were conducted in accordance with the National Health and Medical Research Council of Australia guidelines for the care and use of animals for scientific purposes and were approved by the Monash Institute of Pharmaceutical Sciences Animal Ethics Committee (Protocol MIPS 27467). Female C57BL/6J mice (6–8 weeks old) were acquired from the Monash Animal Research Platform (MARP) and were kept on a 12-h light-dark cycle with food and water available ad libitum. 

To obtain highly purified primary mBECs, the Adult Brain Dissociation kit for mice was utilised and a magnetic-activated cell sorting (MACS^®^) system from Miltenyi Biotech was employed following the manufacturer’s protocols, albeit with some specific optimisations. In brief, brains from two female 6–8-week-old C57BL/6J mice (2 brains per sample, *n* = 6 per experimental group, 12 mice for transport experiments and 12 mice for protein quantification experiments) were removed under sterile conditions and washed with cold HBSS buffer containing 0.33 M pyruvate. The brain was rolled over filter paper to remove the meningeal layers and large blood vessels. The cortex was dissected and then homogenised and placed in a C-tube followed by enzymatic digestion (provided in the kit) while being maintained at 37 °C for 30 min with rotation on the gentleMACS™ Octo Dissociator. The homogenate was then strained using a pre-wet 70 µm MACS smart strainer. The resulting cell suspension was centrifuged at 300× *g* and the pellet was resuspended in 6.2 mL of HBSS buffer and 1.8 mL of cold Debris Removal Solution and centrifuged at 3000× *g* for 10 min at 4 °C. Three separation layers were formed, and the remaining pellet was resuspended in 1× Red Blood Cell Removal solution and incubated in the refrigerator for 10 min. Following the incubation, 10 mL of D-PBS buffer containing 0.5% (*w*/*v*) BSA was added. The resulting solution was centrifuged at 300× *g* for 10 min and the cell pellet was resuspended in a 100 µL solution containing 90 µL of cold DPBS/BSA buffer and 10 µL of CD31-specific mouse MACS^®^ MicroBeads and incubated at 4 °C in the dark for 15 min. The cells were washed with 1 mL D-PBS/BSA buffer and centrifuged at 300× *g* for 5 min retaining the cell pellet. The cells were resuspended in 500 μL of D-PBS/BSA buffer and then subjected to magnetic separation using the MACS^®^ MS columns and a MACS^®^ separator. The column was washed to ensure purity and maximum yield and finally eluted without the magnetic column to collect CD31 positive cells in 900 µL of EBM-2 growth media. The resulting cell suspension was resuspended and plated at a density of 50,000 cells/cm^2^ onto pre-collagenated (Rat-tail collagen Type I) cell culture plates. As an additional purification step, mBECs were then subjected to a 3 μg/mL puromycin (in EBM2 media) treatment for 72 h to eliminate non-endothelial cells. mBECs were maintained at 37 °C in a 5% CO_2_ incubator with media change every two days until 80% confluency was reached. 

Immunocytochemistry (ICC) and fluorescence-activated cell sorting (FACS) were employed to confirm the absence of potentially contaminating neurons, astrocytes or microglia and to determine the percentage of mBECs. Following the isolation, the cell suspension was seeded into a pre-collagenated 8-well ibiTreat (ibidi cells in focus) plate followed by 72 h of puromycin treatment. mBECs were washed twice in PBS and then incubated in EBM2 growth media for 7 days or until approximately 80% confluent. To prepare the cells for ICC, cells were washed three times for 5 min with 200 µL of PBS. The cells were fixed with 4% (*w*/*v*) PFA in PBS for 10 min at room temperature. Excess PFA was washed with 200 µL of PBS three times followed by permeabilisation with 0.1% (*v*/*v*) Triton^®^ X-100 in PBS. 200 µL of blocking solution of 1% (*w*/*v*) BSA in PBS with 0.1% (*v*/*v*) Tween-20 was added for 30 min at room temperature. Following the blocking step, primary antibodies diluted in blocking buffer were co-incubated according to Table 1 overnight at 4 °C. The next day, cells were washed three times for 5 min with 200 µL of PBS followed by incubation with a secondary Alexa Fluor^®^ antibody diluted in blocking buffer (Table 1) and incubated for 60 min protected from light. Following incubation, cells were again washed three times for 5 min with 200 µL of PBS and counter-stained with 4′,6-diamidino-2-phenylindole (DAPI) (25 mg/mL) diluted 1:10,000 for 15 min followed by the final repeated wash step as above. The isolated cells were imaged using the Leica SP8 Lightning confocal microscope (Wetzlar, Germany).

Following seeding and 72 h puromycin treatment, cells were detached with 0.25% (*w*/*v*) trypsin-EDTA and centrifuged at 650× *g* for 5 min to collect the pellet. mBECs were resuspended in a FACS sorting buffer consisting of 1 mM EDTA, 25 mM HEPES (pH 7) and 1% (*w*/*v*) FBS in Ca^2+^ and Mg^2+^ free PBS. The suspension of cells was filtered through a 40 μm mesh strainer to obtain a single-cell suspension. Cells were sorted using the FACS Canto™ II Flow Cytometer (Franklin Lakes, NJ, USA) and gated for cell debris, single cells followed by a viability V450 marker and finally the phycoerythrin (PE) CD31+ to detect the percentage yield and purity of mBECs isolated using the MACS^®^ system. Controls for the correct detection of the antibodies specific to viability V450, fluorescein isothiocyanate (FITC) CD45+ (peripheral cells) and allophycocyanin (APC) CD11b+ (microglia) were sampled concurrently with PE CD31+. 

### 2.3. Treatment of mBECs with Cu(ATSM) to Assess P-gp Expression and Function

Once 80% confluency was reached, mBECs were treated with either 100 nM Cu(ATSM) or vehicle (0.1% (*v*/*v*) DMSO) for 24 h prior to being lysed for Western blot (WB) analysis or for P-gp functional studies as previously described in our laboratory [40]. Uptake studies were conducted using a fluorescent P-gp substrate R123. In brief, accumulation of R123 in mBECs was measured to ascertain P-gp efflux capacity following a pre-incubation of 5 μM R123 in transport buffer (10 mM HEPES in HBSS pH 7.4) for 60 min (Section 3.2 schematic). Following incubation, cells were lysed with 100 μL of 1% (*v*/*v*) Triton^®^ X-100 on a plate shaker at 4 °C for 20 min. 50 μL of R123 standards (prepared in 1% (*v*/*v*) Triton^®^ X-100 in milliQ water) and 50 µL of the lysate sample from each well were then transferred into a 96-well plate. Fluorescence was detected using the Enspire fluorescence spectrophotometer at excitation and emission wavelengths of 511 and 534 nm, respectively. The fluorescent R123 (nmol) signal was normalised to total cellular protein determined using the Pierce™ BCA protein assay (mg) and the accumulation (nmol/mg) was compared between vehicle and Cu(ATSM)-treated cells.

### 2.4. Dosing of Cu(ATSM) to C57BL/6J Mice

The dosing regimen of Cu(ATSM) was selected based on disease-modifying doses of 30 mg/kg in disease models of amyotrophic lateral sclerosis and Parkinson’s disease, demonstrated to not have any adverse side effects [45,46,47]. Female C57BL/6J mice (6–8 weeks old), randomly allocated to a treatment or vehicle group, were orally administered a standard suspension vehicle (SSV) consisting of 0.9% (*w*/*v*) NaCl, 0.5% (*w*/*v*) Na-carboxymethylcellulose, 0.5% (*v*/*v*) benzyl alcohol, and 0.4% (*v*/*v*) Tween-80 with or without 30 mg/kg of Cu(ATSM). Following daily oral dosing for 28 days, the mice were humanely killed, and their brains were removed to isolate microvessels and the small intestine, liver and kidneys were isolated for P-gp expression and metallomic analysis, or the mice were anaesthetised for functional P-gp functional studies.

### 2.5. Brain Microvessel Membrane Fraction Isolation and Organ Harvesting

After completing the dosing regimen, mice were anesthetised with 1–5% isoflurane and then euthanised by cervical dislocation. Brains were removed and carefully rolled over filter paper to remove the meningeal layers and larger surface blood vessels. The olfactory bulb, cerebellum and brain stem were dissected out and only the cerebral cortices of mice (vascularised region representing the BBB) were included in the isolation. In order to obtain enough protein for quantification of P-gp using WB, 3–4 mouse cortices were combined to obtain enough microvessel enriched fractions (MEF) using a modified protocol previously reported by our laboratory (*n* = 6, 24 Vehicle (SSV) and 24 Cu(ATSM) (30 mg/kg) group) [48]. Briefly, the pooled brains were homogenised in 10 mL of DMEM GlutaMAX™ using a pre-cooled Dounce homogenizer (Tissue Grinder, Potter-ELV, Wheaton Industries, Millville, NJ, USA) with 10 vertical strokes and 1–2 twisting motions per vertical stroke. The homogenate was centrifuged at 2000× *g* at 4 °C for 5 min to obtain a pellet, and the pellet was resuspended to a final concentration of 15% (*w*/*v*) BSA in DMEM GlutaMAX™ and further centrifuged at 2000× *g* at 4 °C for 30 min. After centrifugation, the resulting supernatant (brain parenchymal fraction) and pellet (MEF) were separated, and the MEF pellet was rinsed with DMEM GlutaMAX™ to remove excess BSA before being filtered through a 100 μm nylon mesh strainer. The suspension was then centrifuged to obtain a pellet that was rinsed twice with PBS, snap-frozen in liquid nitrogen and stored at −80 °C for future membrane fraction isolation. Small intestines, kidneys, and livers of Cu(ATSM) and vehicle-dosed mice were also dissected, snap-frozen, and stored at −80 °C for later use for P-gp protein quantification or inductively-coupled plasma mass spectrometry (ICP-MS) for metallomic analysis. 

To determine a more accurate representation of the expression of the functional membrane-bound ATP-binding cassette transporter (rather than investigate total cellular P-gp), membrane fractions of the isolated MEF samples were obtained according to the Mem-PER^TM^ Plus Membrane Protein Extraction kit manufacturer’s protocol, albeit with minor modifications. In brief, the capillary pellet isolated from the MEF were thawed on ice and resuspended in 500 μL of cell permeabilisation buffer provided in the kit with cOmplete^™^ mini protease inhibitor, transferred to a 1.5 mL Eppendorf tube and incubated at 4 °C for 10 min with constant gentle agitation. The permeabilised samples were centrifuged at 16,000× *g* for 15 min at 4 °C. The supernatant containing the cytosolic fractions were separated and aliquoted for protein determination and cytosolic metallomic analysis via ICP-MS. The remaining pellet was resuspended in 500 μL of membrane solubilisation buffer provided in the kit with cOmplete™ mini protease inhibitor and incubated at 4 °C for 30 min under gentle agitation. Following incubation, the solubilised membrane fractions were centrifuged at 16,000× *g* for 15 min at 4 °C. The supernatant containing membrane fraction proteins was collected and aliquoted for WB analysis. 

### 2.6. ICP-MS of Brain MEFs and Organ Homogenates

The cytosolic fractions (250 μL) of microvessels, as well as peripheral tissue samples (small intestines, liver and kidneys) isolated from Cu(ATSM) (30 mg/kg) and vehicle-treated mice (*n* = 6–8) were lyophilised prior to sample preparation for ICP-MS. The wet weights of organs were determined prior to lyophilisation. Small intestines were approximately 150 mg, livers were approximately 150 mg, and the two kidneys had a combined weight of approximately 250 mg. Nitric acid (HNO_3_) (65% Suprapur, Merck, Darmstadt, Germany) was added to each sample and digested overnight. The samples were further digested at 90 °C for 20 min in a heating block and then an equivalent volume of hydrogen peroxide 30% (*v*/*v*) (Aristar, BDH) was added to each sample, which was allowed to digest for 30 min. Samples were allowed to stop effervescing for 30 min before being heated again for 15 min at 70 °C. The average reduced volume was determined, and 50 μL aliquots of samples were further diluted 1:20 for MEF cytosolic fractions and 1:400 for tissue samples with 1% HNO_3_ (*v*/*v*). The ICP-MS measurements were made using an Agilent 7700 series ICP-MS instrument (Santa Clara, CA, USA) with routine multi-element operating conditions using a Helium Reaction Gas Cell. The instrument was calibrated using 0, 5, 10, 50, 100 and 500 ppb of certified multi-element ICP-MS standard calibration solutions (ICP-MS-CAL2-1, ICP-MS-CAL-3 and ICP-MS-CAL-4, Accustandard, New Haven, CT, USA) for a range of elements. Additionally, a certified standard solution containing 200 ppb of Yttrium (Y89) was used as an internal control (ICP-MS-IS-MIX1-1, Accustandard). The elements assessed included sodium, magnesium, phosphorous, calcium, iron, cobalt, nickel, copper, zinc, selenium, and rubidium, and elements with abundances at or below detection limits were excluded from the data analysis.

### 2.7. WB of Brain MEFs and Organ Homogenates

An aliquot of the cytosolic and membrane fractions (10 μL) obtained from the isolated MEFs was quantified for total protein using the BCA protein assay against known standards of BSA. For the small intestine (jejunum), liver (left lobe) and kidney (left kidney), approximately 20 mg of wet-weight tissue was dissected from each main sample and placed into a 4 mL homogenisation tube containing 0.5 mL of Pierce IP lysis buffer and 7 × concentrated protease inhibitor solution (1:6 ratio). The tissues were briefly homogenised for 10 s per tube using an IKA T25 digital ULTRA-TURRAX tissue homogeniser (Lab Gear Australia, Milton, Queensland, Australia) set at 4000/min. The samples were then left on ice for 30 min, centrifuged at 15,000× *g* for 15 min, and the supernatant was collected. The supernatant was then aliquoted for protein content analysis using the BCA protein assay and stored at −80 °C until further use.

Following protein quantification, the appropriate volume to obtain a consistent 20 μg protein load of cytosolic and membrane fractions as well as tissue lysates was mixed with 6 × Laemmli buffer in a 5:1 ratio and incubated at 37 °C for 20 min. Electrophoresis was carried out on Mini-Protein^®^ TGX^™^ Precast gels (4–15% acrylamide) at 100 V for 30 min and then 150 V for 60 min. Gels, extra thick blot paper, and 0.45 μm pore-sized nitrocellulose membranes were equilibrated in a transfer buffer containing 20% (*v*/*v*) methanol for 20 min before transfer. The transfer was executed using the Bio-Rad Trans-Blot Turbo transfer system (Hercules, CA, USA) set to 40 min at 25 V and 1.0 A. Following a brief rinse in TBS-T, membranes were incubated in Intercept^®^ blocking buffer at room temperature for 1 h, rinsed again in TBS-T, and then incubated with a 1:500 dilution of the primary C219 monoclonal antibody for P-gp and a 1:500,000 dilution of the primary mouse antibody for β-actin (for peripheral tissues and cytosolic fractions of MEFs) or a 1:25,000 dilution of the Na/K ATPase membrane loading control antibody (for membrane fractions of MEFs) in TBS-T overnight at 4 °C. After four × 5 min membrane washes in TBS-T, the membranes were incubated with secondary IRDye^®^ 800CW goat anti-mouse antibody (1:7500) and IRDye^®^ 680LT donkey anti-rabbit antibody (1:20,000) for 2 h at room temperature followed by another four × 5 min washes. Membranes were scanned on the GE Amersham™ Typhoon™ instrument (Chicago, IL, USA). Densitometric analysis was performed via the ImageJ software Version 1.53t (National Institutes of Health, Bethesda, MD, USA) and the data were normalised to the β-actin or Na/K ATPase signal and expressed as relative fold-changes compared to controls.

### 2.8. In situ Transcardiac Brain Perfusion to Assess BBB Transport

An in situ transcardiac brain perfusion technique was utilised to evaluate the transport of the P-gp substrate [3H]-digoxin across the BBB in vehicle and Cu(ATSM)-treated mice (*n* = 6–7) (Section 3.4. schematic), following a previously described protocol from our laboratory [49]. [14C]-sucrose permeability across the BBB was determined to ensure vascular integrity remained intact following the Cu(ATSM) treatment. Ketamine (133 mg/kg) and xylazine (10 mg/kg) were used to establish surgical anaesthesia of mice (*n* = 6–7 per treatment group), and the mice were kept warm on a surgical platform during the procedure. The thoracic cavity was exposed, and the descending aorta was ligated to restrict blood flow to peripheral organs and both jugular veins were severed immediately prior to perfusion. A pre-warmed (37 °C) Krebs bicarbonate ringer buffer (128 mM NaCl, 4.2 mM KCl, 1.5 mM CaCl_2_, 0.9 mM MgSO_4_, 24 mM NaHCO_3_, 2.4 mM NaH_2_PO_4_, and 9.0 mM glucose carbogenated with 95% O_2_ 5% CO_2_, adjusted to pH 7.4) containing [3H]-digoxin (0.1 µCi/mL) and [14C]-sucrose (0.05 µCi/mL) was injected (25G butterfly needle) into the left ventricle of the heart at a rate of 5 mL/min for 2 min using a Harvard infusion pump (Harvard Apparatus, Holliston, MA, USA). After 2 min, the perfusion was halted followed by cervical dislocation and the brain was collected in liquid scintillation vials and digested in 2 mL of Solvable™ at 50 °C overnight. The following day, colour was neutralised by adding 200 µL of hydrogen peroxide (30% *v*/*v*). The brain sample was mixed with 10 mL of Ultima Gold scintillation cocktail, while 2 mL of Ultima Gold scintillation cocktail was added to 100 µL of the radioactive perfusion fluid to quantify the amount of probe in the spike solution. The radioactivity of each sample was counted using a PerkinElmer 2800TR liquid scintillation analyser (Waltham, MA, USA). The apparent brain distribution volume of the probe compound (brain:perfusate ratio, mL/g) was determined based on previously described methods [49]. The apparent tissue distribution volume of the [3H]-digoxin (B:P, mL/g) was calculated using Q_brain_/C_p_ normalised by brain weight (g), where Q_brain_ is the radioactivity in the brain (DPM/g) (radioactivity from the vascular volume subtracted), and C_p_ is the radioactivity per mL of perfusate (DPM/mL). The vascular volume was determined using the B:P of [14C]-sucrose.

### 2.9. Statistical Analyses 

All figures and data were generated and analysed using GraphPad Prism^®^ software version 8.1.1 (GraphPad Software Incorporated). All data were expressed as mean ± SEM, where all replicates compared were biological replicates. When comparing between two groups, a student’s unpaired *t*-test was performed. Levels of significance are indicated for individual experiments, with differences considered to be statistically significant when the *p*-value was less than 0.05 (*p* < 0.05). The number of biological replicates of individual animals or pooled samples are represented by *n* (i.e., *n* = 1 for each pooled sample). Data were assessed for normality using the Sharpiro–Wilk test and all data points were used for statistical analysis. There were no inclusion or exclusion criteria pre-determined, and no blinding was performed, however, animals selected for treatment groups were randomised to allocated groups in the study.

## 3. Results

### 3.1. Characterisation and Purity of Isolated Primary mBECs

**Figure 1:**
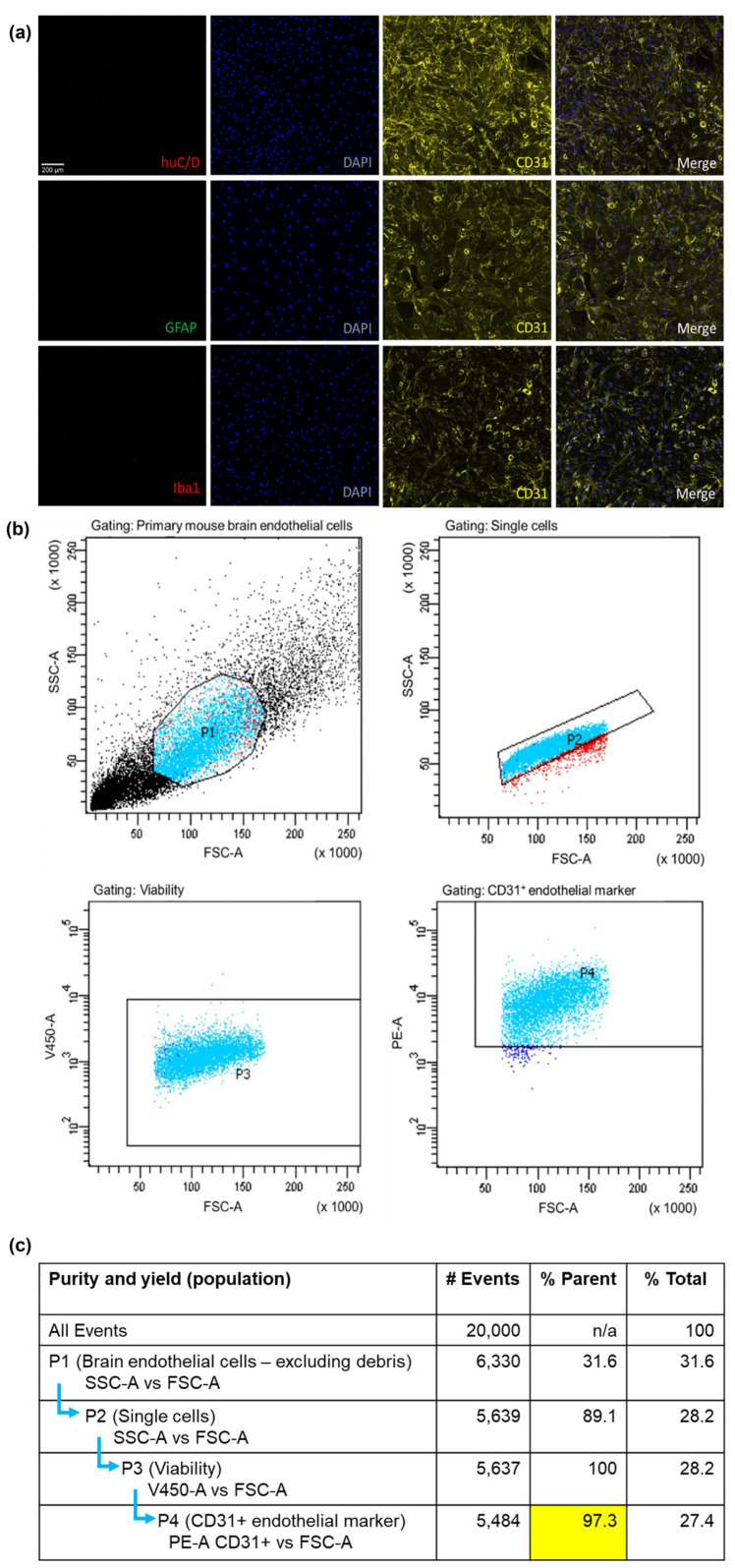
The MACS^®^ isolation method yields pure endothelial cells. Primary mBEC purity and yield assessed by ICC (**a**) indicating no neuronal (huC/D), astrocyte (GFAP) or microglial (Iba1) contamination but positive confirmation of CD31+ brain endothelial cells. Each sample consists of 2 brains pooled from C57BL/6J mice. A 97.3% CD31+ brain endothelial specific isolation was acquired that was gated (selection of cells indicated by light blue dots) for cell debris, single and viable cell populations confirmed by FACS (**b**). Table (**c**) indicates the gating strategy for FACS with forward scatter area (FSC-A) indicating cell size, side scatter area (SSC-A) to the granularity of the cells, viability 450 marker area (V450-A), and phycoerythrin fluorophore CD31+ marker (PE-A CD31+).

To validate the purity and yield of primary mBECs isolated with the adapted MACS^®^ isolation method, a qualitative and quantitative approach was utilised. ICC was employed to assess if other CNS cells such as neurons, astrocytes and microglia remained in the suspension of cells after the magnetic separation of CD31-positive endothelial cells. It was clearly observed that there was an abundance of CD31-positive endothelial cells (i.e., mBECs) with no other contaminating cells present following the isolation and 72 h puromycin treatment (Figure 1a). FACS also confirmed that the cell suspension from the isolation method consisted of CD31-positive endothelial cells of 97.3% purity after the sample was gated for cell debris, single-cell suspension and viability (Figure 1b). 

### 3.2. Cu(ATSM) Increases P-gp Expression and Function in Isolated Primary mBECs

**Figure 2:**
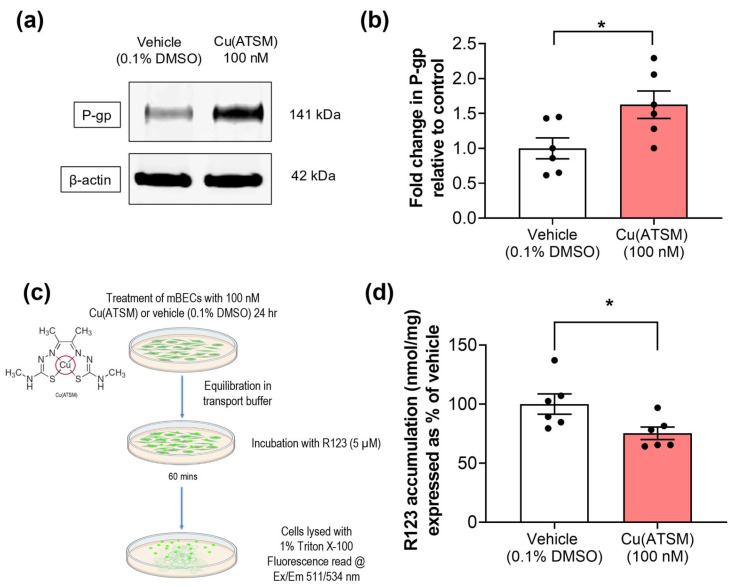
Primary mBECs treated with 100 nM Cu(ATSM) show increased expression (**a**,**b**) compared to mBECs treated with a vehicle (0.1% DMSO) for 24 h. (**a**) Representative WB of expression levels of P-gp and β-actin housekeeping protein from one gel. (**c**) P-gp function was assessed by measuring the cellular accumulation of a fluorescent substrate, R123 (nmol), normalised to total protein (mg). (**d**) Cu(ATSM) reduces accumulation of R123 in mBECs following a 24 h treatment. Data are presented as mean ± SEM (*n* = 6, independent cell culture isolations, 2 brains per pooled sample) expressed as fold-change for (**b**) WB or (**d**) % of vehicle (0.1% DMSO) for accumulation of R123 (nmol/mg protein) with * *p* < 0.05 when compared to the vehicle-treated group, assessed by an unpaired t-test. Illustration created with BioRender.com.

Primary mBECs treated with 100 nM Cu(ATSM) for 24 hrs exhibited a 1.6-fold increase in P-gp expression compared to vehicle (0.1% DMSO)-treated cells (Figure 2a,b). The functionality of P-gp efflux was evaluated using a fluorescence-based assay with a known P-gp substrate, R123. A 25% reduction in the accumulation of R123 was observed in mBECs treated with Cu(ATSM) compared to vehicle (0.1% DMSO)-treated mBECs (Figure 2d), which was in line with the increase in P-gp expression.

### 3.3. Mice Dosed with Cu(ATSM) Show Increased P-gp Abundance and Levels of Cu in Isolated Brain MEFs

**Figure 3:**
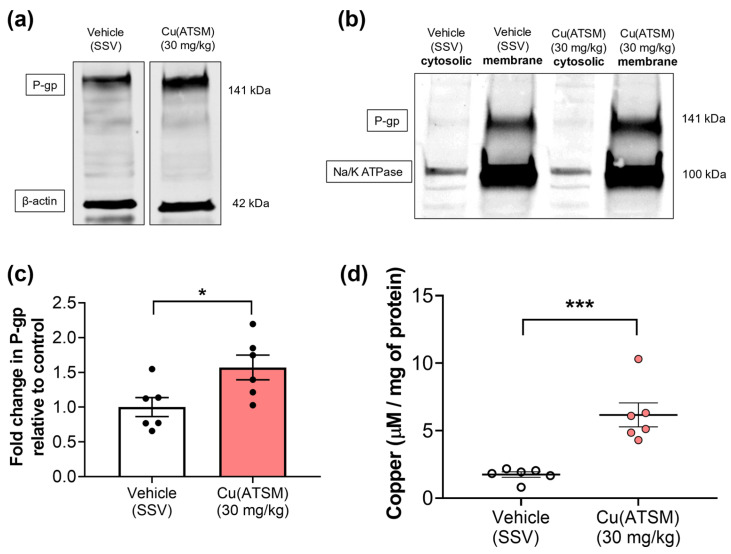
Cu(ATSM) upregulates P-gp expression with an increase in cytosolic copper (Cu) in MEFs isolated from mouse brain. Representative WB of expression levels of (**a**) P-gp and β-actin in MEF lysates and (**b**) P-gp and Na/K ATPase in MEF cytosolic & membrane fractions. A graphical representation of (**c**) P-gp expression as assessed by densitometry of membrane fractions and (**d**) levels of Cu in cytosolic MEFs from vehicle (SSV) or Cu(ATSM) (30 mg/kg)-treated mice. Data are presented as mean ± SEM (*n* = 6, 4 pooled brains per sample) expressed as fold change of vehicle (SSV) for WB or levels of Cu (μg/mg of protein) for ICP-MS with * *p* < 0.05, *** *p* < 0.001, when compared with the vehicle-treated group, assessed by an unpaired *t*-test.

To assess if the changes observed at the cellular level (mBECs) and from our previous studies (in hCMEC/D3 cells) were able to be translated into an in vivo system, mice were dosed daily with 30 mg/kg of Cu(ATSM) via oral administration over 28 days. When compared with mice dosed with vehicle (SSV), there was a visible increase in P-gp abundance in brain MEFs isolated from Cu(ATSM)-treated mice that were investigated in a pilot study to assess potential changes in expression (Figure 3a). Following this observation, all other samples were processed to assess the membrane fractions and cytosolic fractions for P-gp expression to account for functional membrane-bound P-gp. The levels of P-gp detected in the cytosolic fractions were negligible, whereas MEFs from the Cu(ATSM)-treated mice exhibited a 1.5-fold increase in membrane-bound P-gp when compared to those obtained from the vehicle (SSV)-treated group (Figure 3b,c). This correlated with an increase in the levels of Cu in the brain MEFs from the Cu(ATSM) treated mice (6.2 ± 0.88 μM/mg protein) compared to the brain MEFs isolated from vehicle-treated mice (1.7 ± 0.20 μM/mg protein) (Figure 3d).

### 3.4. Mice Dosed with Cu(ATSM) Show Increased BBB Function of P-gp

**Figure 4:**
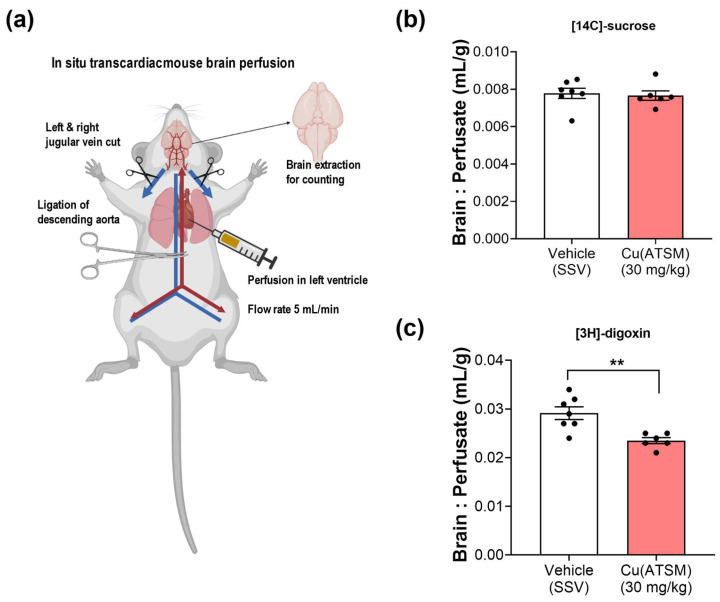
Cu(ATSM) enhances P-gp-mediated efflux function. In situ transcardiac brain perfusion technique (**a**) demonstrates an intact, uncompromised BBB with no difference in the brain:perfusate (B:P) ratio of [14C]-sucrose (**b**) but a decreased B:P ratio of the P-gp substrate [3H]-digoxin (**c**) in Cu(ATSM)-treated (30 mg/kg daily for 28 days) mice compared to the SSV-treated group. Data are presented as mean ± SEM (*n* = 6–7) expressed as brain: erfusate (mL/g) with ** *p* < 0.01 when compared with the SSV-treated group, assessed by an unpaired *t*-test. Illustration created with BioRender.com.

A clinically relevant substrate for P-gp, digoxin, was utilised to assess the efflux capacity of P-gp and to ascertain whether enhanced P-gp expression leads to enhanced efflux function in mice treated with Cu(ATSM). The in situ transcardiac brain perfusion studies demonstrated a 20% decrease in [3H]-digoxin uptake into the brains of mice treated with Cu(ATSM) (0.023 ± 0.00062 mL/g) compared to the vehicle-treated group (0.029 ± 0.0013 mL/g) (Figure 4). It was important to assess whether BBB integrity was maintained following the 28-day daily dosing of Cu(ATSM), and this was confirmed by assessing the transport of a paracellular diffusion marker [14C]-sucrose, which also provided a value of the microvascular volume. There was no significant difference observed in the cerebral vascular volume of [14C]-sucrose between the Cu(ATSM) (0.0077 ± 0.00025 mL/g) and vehicle-treated group (0.0078 ± 0.00027 mL/g) (Figure 4).

### 3.5. Cu(ATSM) Treatment Increases Copper and P-gp Expression in a Peripheral Organ-Specific Manner

**Figure 5:**
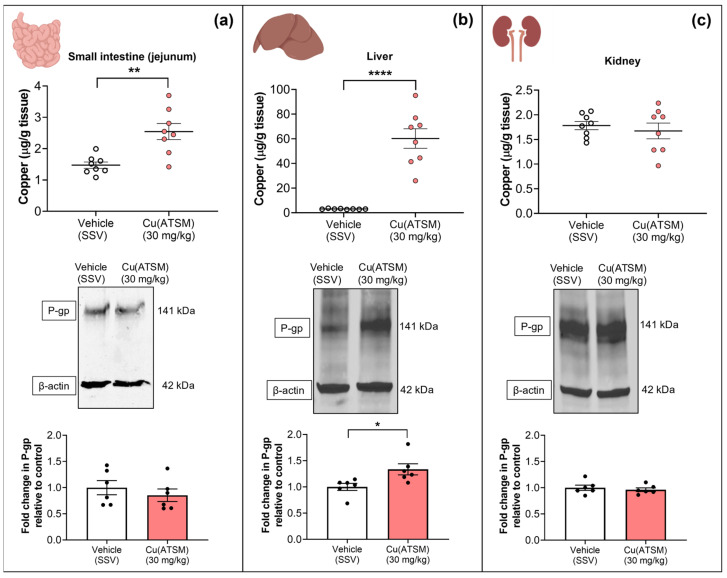
Cu levels detected in peripheral organs measured by ICP-MS following daily dosing of either Cu(ATSM) (30 mg/kg) or vehicle (SSV) for 28 days to C57BL/6J mice. Cu(ATSM) increased Cu in the small intestine (jejunum) (**a**) and liver (**b**) but not in the kidneys (**c**). P-gp protein expression was enhanced in the liver tissue only (**b**). Representative WBs of expression levels of P-gp and β-actin of peripheral organs and graphical representation of P-gp expression as assessed by densitometry (bottom). Data are presented as mean ± SEM (*n* = 6–8) as Cu levels (μg/g of tissue wet weight) or a fold change in P-gp compared to vehicle (SSV) with * *p* < 0.05, ** *p* < 0.01, **** *p* < 0.0001 when compared to the SSV-treated group as assessed by an unpaired *t*-test. Illustration created with BioRender.com.

To assess the impact of Cu(ATSM) on P-gp expression in peripheral organs and the biodistribution of Cu following the dosing regimen, WB and ICP-MS were utilised (Figure 5 & Table 2). The mice dosed with 30 mg/kg Cu(ATSM) exhibited a significantly higher Cu level in the jejunum (2.5 ± 0.26 μg/g tissue) when compared to vehicle-treated mice (1.4 ± 0.10 μg/g tissue), but this was not associated with a change in P-gp expression (Figure 5a). The biggest change in treatment-associated Cu levels was observed in the liver where Cu(ATSM)-treated mice had 60.2 ± 7.89 μg/g tissue Cu compared to 3.0 ± 0.12 μg/g tissue Cu in vehicle-treated mice (a 19.9-fold difference) (Figure 5b) and this was accompanied with a 1.3-fold increase in P-gp expression. No signs of liver toxicity or damage were observed in the tissues collected for analysis. Interestingly, there was no change in the levels of Cu nor P-gp expression in the kidneys isolated from the vehicle and Cu(ATSM)-treated mice. 

The analysis of the levels of other metals in the MEF and peripheral organs revealed no significant differences between the two treatment groups except within the liver, as summarised in Table 2. With regards to the liver, Cu(ATSM)-treated mice exhibited a significant reduction of between 0.8–0.9-fold in magnesium, phosphorus, potassium, and iron. However, there was a 1.3-fold increase in zinc (Zn) in the livers of Cu(ATSM)-treated mice (Table 2).

## 4. Discussion

Although P-gp impedes drug delivery across the BBB, it is also involved in the clearance of neurotoxic Aβ peptides from the brain, making it a crucial contributor to AD etiology [24,26,29]. The identification of potential modulators of P-gp expression and function, and understanding the regulatory mechanisms involved in maintaining the function of this gatekeeper, has numerous applications in developing neurotherapeutics. Copper influences pathways involved in P-gp regulation, and our previous findings showed modulation of key efflux transporters in hCMEC/D3 cells treated with copper-modulating compounds [36,40,50,51]. Therefore, this study investigated the impact of Cu(ATSM) in mBECs and the mouse microvasculature in vivo to determine if the observed changes in hCMEC/D3 cells translated to mice, where Cu(ATSM) has been shown to exhibit disease-reversing effects. Cu(ATSM) has a relatively stable structure compared to other bis(thiosemicarbazone) counterparts due to the presence of two additional methyl groups which increase the reduction potential and thus prevent the release of Cu [52]. However, under hypoxic conditions where the reducing capacity of the intracellular environment is increased, Cu(ATSM) is reduced, leading to an increase in bioavailable Cu. This selective property makes Cu(ATSM) a potential therapeutic agent for cells and tissues under oxygen-deprived stress in various disease states [53]. Cu(ATSM) has great potential to be utilised in diagnostics and has recently emerged as a promising pharmacotherapeutic for the treatment of various neurodegenerative disorders including amyotrophic lateral sclerosis, Parkinson’s disease, ischemic stroke and neuroinflammation [45,54,55,56,57]. While Cu(ATSM) has been designed to release Cu in hypoxic environments, we demonstrated that this entity also increases Cu in hCMEC/D3 cells, an outcome associated with an increase in P-gp expression and function. This suggests that in addition to disease-modifying effects, Cu(ATSM) may have beneficial effects at the BBB, which has not been previously investigated. 

This study demonstrated the successful isolation of 97.3% pure mBECs using the MACS^®^ system and this was validated using both ICC and FACS. The purity of mBECs was comparable to other studies using other techniques for isolation such as glass bead purification, or BSA or dextran density filtration without magnetic separation, where an 87–95% purity was obtained as confirmed by FACS, with the majority of contaminating cells reported to be microglia or astrocytes [58,59,60]. The MACS^®^ system proved to be a significant advance for isolating mBECs due to the commercially available complete kits that facilitated the relatively fast, reliable, and relatively simple acquisition of pure endothelial cells. The purity of cells was essential to investigate the effects of Cu(ATSM) on the expression and function of P-gp so that we could be confident that any alterations observed were indeed mBEC-specific given that P-gp is also expressed in astrocytes, pericytes and microglia [61,62]. The increase in P-gp expression and function in mBECs following Cu(ATSM) treatment was comparable to that found in hCMEC/D3 cells previously in our laboratory, where a 24 h Cu(ATSM) treatment at the same concentration (100 nM) lead to a 2-fold increase in P-gp expression and 30% increase in efflux of R123 [40]. Other studies have used alternative methods to enhance P-gp expression and function such as activation of the pregnane X receptor (PXR) as well as blocking P-gp degradation [24,63,64,65]. Expression and function of P-gp were significantly enhanced in all of these studies compared to controls, but direct comparison of P-gp function cannot be made as the methods to assess transport ranged from cellular transport of diverse P-gp substrates to isolated capillary transport assays, and thus no direct comparison to in situ brain perfusion studies could be made. However, taken together, these studies demonstrate that P-gp can be modulated with various interventions and similar to other approaches, Cu(ATSM) has the potential to modulate the trafficking of agents into and out of the brain.

The mechanism of P-gp upregulation following Cu(ATSM) treatment was not explored in this study but our previous studies have elucidated a potential pathway in hCMEC/D3 cells that is likely replicated in mBECs. The regulation of MDR1 transcription and subsequent P-gp expression has been associated with the activation of the mitogen-activated protein kinase (MAPK) pathway and downstream activation of ERK1/2 [66,67,68]. Studies, including our previous findings, have demonstrated that Cu(ATSM) activates this pathway, leading to the phosphorylation of ERK1/2 [40,69,70,71]. By inhibiting this pathway with U0126 (an upstream inhibitor of mitogen-activated protein kinase kinase 1/2), the beneficial effects of Cu(ATSM) are prevented [40]. As these pathways are also present in mBECs [72,73], it is likely that Cu(ATSM) activates these pathways to increase P-gp expression. Cu(ATSM)-mediated upregulation of P-gp may also be attributed to the activation of the neuroprotective molecular sensor of oxidative stress, nuclear erythroid 2–related factor 2 (Nrf2). Nrf2 was shown to promote P-gp expression and transport activity in rat brain capillaries and this potential pathway has been demonstrated to be activated by Cu(ATSM) [69,74,75]. While Nrf2 is a ligand-activated transcription factor, it has been suggested that Nrf2-mediated P-gp upregulation occurs indirectly, involving activation of NF-κB via p53 and p38 signalling [74]. Studies in murine models of traumatic brain injury have demonstrated that Nrf2 ligand administration is neuroprotective and helps to maintain BBB integrity [76]. Srivastava et al. (2016) demonstrated with both oral delivery of Cu(ATSM) in mice and in vitro that Cu(ASTM) treatment leads to a significant upregulation of Nrf2-dependent antioxidant defences that elicit the cardioprotective effects of Cu(ATSM) [69]. Furthermore, ERK1/2 activation has been shown to mediate Nrf2 phosphorylation [77], thus providing an integrated mechanism through which Cu(ATSM) may enhance Nrf2 signalling. Taken together, this suggests a potential mechanism of action for P-gp regulation in response to Cu(ATSM) suggesting that it may be mediated through the activation of, but not limited to MAPK, ERK1/2 and Nrf2 signalling. Further studies are needed to elucidate the exact mechanisms and pathways involved in the regulation of P-gp in mice following treatment with Cu(ATSM). 

While our in vitro studies in mice and human brain endothelial cells were complementary, it was important to assess whether Cu(ATSM) had a similar P-gp-enhancing effect *in vivo*. Mice dosed with Cu(ATSM) over 28 days showed a 1.5-fold increase in P-gp expression in brain MEFs. The MEFs isolated from mice were separated into membrane and cytosolic fractions to probe for membrane associated-P-gp which represents the functional ATP-binding cassette efflux transporter (Figure 3b). The cytosolic fractions did not show P-gp bands after completing the WB indicating that the majority of detected P-gp was indeed the membrane-associated functional protein. The enhanced expression was accompanied by a 20% reduction in the transport of the P-gp substrate [3H]-digoxin. The brain:perfusate ratio of [3H]-digoxin was significantly reduced in Cu(ATSM) treated mice indicating enhanced P-gp mediated efflux capacity, and this ratio was comparable (~0.002–0.003 mL/g) to ratios found in other studies in our laboratory that used a 150-day old mouse model of amyotrophic lateral sclerosis [78]. This comparison suggests that P-gp-mediated [3H]-digoxin efflux is similar across different ages and strains of mice. To ensure that Cu(ATSM) did not compromise the integrity of the BBB and thus alter paracellular transport, [14C]-sucrose was used as an integrity marker. There was no significant difference in [14C]-sucrose transport between the treatment groups and we thus concluded that Cu(ATSM) does not change paracellular permeability. The B:P ratio of [14C]-sucrose was similar to that found in recent studies for vascular volume determination in transcardiac brain perfusion studies (ranging from 0.006 mL/g–0.013 mL/g) [49,78,79]. The findings of the current study demonstrate that Cu(ATSM) may be a promising drug candidate for increasing P-gp expression and function in vivo, and may represent a potential strategy to enhance clearance of Aβ across the BBB, with possible disease-modifying effects. 

Having shown that Cu(ATSM) increased the BBB expression and function of P-gp, we then assessed the levels of Cu (μg/mg protein) being delivered to brain MEFs following daily oral administration of 30 mg/kg Cu(ATSM) for 28 consecutive days. To our knowledge, this is the first analysis of MEF metal levels in mice treated with Cu(ATSM) (Table 2). Using ICP-MS, a 3.5-fold increase in Cu was detected in the cytosolic MEFs from Cu(ATSM)-treated mice when compared to vehicle-treated mice. This finding was consistent with our previous in vitro studies showing a significant increase in intracellular Cu, supporting the hypothesis that Cu(ATSM) increases both bioavailable Cu and P-gp expression in vivo as well as in vitro [40]. As was observed with our studies in hCMEC/D3 cells, Cu(ATSM) treatment did not result in any detectable differences in the levels of other metal levels in the brain MEF. Interestingly, brain Cu levels have been reported to increase 1.2-fold in mice treated with clioquinol [80], an agent that we have shown increases P-gp expression in hCMEC/D3 cells [36]. It would therefore be expected that clioquinol would also increase P-gp expression in brain microvessels, however, we have shown that neither P-gp expression nor Cu levels in MEFs are significantly different between vehicle and clioquinol-treated mice [50]. This is an important distinction to make as it suggests that compounds need to modulate brain microvascular Cu levels specifically, rather than elevate Cu levels across the brain overall, in order to modulate P-gp expression, as we have demonstrated with Cu(ATSM). 

To obtain a better understanding of the potential systemic effects of Cu(ATSM), we conducted a metallomic analysis of peripheral organs, investigating changes to metal biodistribution as well as P-gp expression in the small intestines, liver, and kidneys of mice treated with oral Cu(ATSM) in comparison to vehicle-treated mice (Figure 5 and Table 2). These organs abundantly express P-gp and although the blood-testis barrier and the placenta were not investigated as part of the current study, future studies could explore the impact of Cu(ATSM) on P-gp that also has a protective role in these tissues. Within the small intestines, there was a significant increase in Cu levels detected but no differences to other metals, and no changes in P-gp expression. While Cu levels are increased in the small intestine, it is possible that the suggested signalling pathways mediating P-gp regulation by Cu in the brain microvessels may not be impacted by Cu in the small intestine, or such pathways may have less of an impact on P-gp regulation in the small intestine. Remarkably, there was a 19.9-fold increase in liver levels of Cu in mice treated with Cu(ATSM) relative to vehicle-treated mice. Compared to the small intestines, the highly vascularised nature of the liver may account for the accumulation of Cu(ATSM) in hepatic tissue. This substantial increase in hepatic Cu levels was accompanied by an increase in P-gp expression. This effect of Cu(ATSM) has the potential to lead to an increase in hepatic P-gp-mediated clearance and therefore it is possible that Cu(ATSM) may affect the pharmacokinetics of other drugs that are P-gp substrates, leading to drug-drug interactions [81]. Interestingly, and only within the liver, a 1.3-fold increase in Zn with Cu(ATSM) treatment was also observed, alongside significant reductions in magnesium, phosphorus, potassium and iron. The reduction in other metals may be attributed to the higher levels of Cu in the liver which may displace these metals, as has been shown in the case of iron mobilisation [31,32]. The increase in Zn is in contrast with what has been reported previously, where an increase in Zn reduces the levels of Cu in the liver because Zn hinders the absorption of Cu and competes with it to bind to the same metal binding sites on proteins [82,83]. However, this would not be expected in this scenario given that it is unlikely that Zn would impact the passive diffusion of Cu(ATSM) into hepatocytes, the agent responsible for the increase in hepatocellular Cu. Furthermore, the kidneys did not exhibit any differences in Cu levels nor P-gp expression. A recent study also reports that following oral administration of Cu(ATSM), no significant changes were observed in levels of Cu in the kidney, but significant increases were observed in the liver and brain which aligns with our observations [84]. However, our study is the first that demonstrates increased Cu availability in the brain microvasculature and the first to associate the increase in Cu levels with an increase in P-gp expression. 

The limitations of the sampling used in this investigation were recognised and acknowledged. The assays employed, namely ICP-MS and WB, were performed on homogenised whole tissue samples and not on sub-organ regions of those tissues other than the small intestines where only the jejunum was analysed. This may limit the ability to detect any region-specific or localised changes in the tissues analysed. The ICP-MS detects total Cu and cannot distinguish between bioavailable and complex-bound Cu. However, the data presented in this study provides a basis for drawing appropriate conclusions and comparisons between treatment groups. The present study paves the way for further research into the potential clinical applications of Cu(ATSM) and similar compounds that target P-gp expression and function at the BBB and offers hope for the development of more effective drug therapies for neurodegenerative diseases such as AD.

## 5. Conclusions

This study has successfully demonstrated the capacity of Cu(ATSM) to enhance P-gp expression and function in mBECs, as well as in vivo, given that oral administration of Cu(ATSM) resulted in increased levels of Cu and P-gp expression in brain MEFs, as well as in the liver. The implications of this research are significant, as they demonstrate the potential of Cu(ATSM) to modulate BBB transport of P-gp substrates, including many drugs as well as Aβ, the toxic peptide accumulating in the brain in AD. The outcomes of this study have provided a foundation for future research and investigation of Cu(ATSM) and its potential as a therapeutic approach for reducing Aβ levels in AD. Further studies are needed to explore the specific mechanism by which Cu(ATSM) upregulates P-gp and investigate the potential of this antioxidative, anti-inflammatory and neuroprotective compound to reduce the burden of Aβ in AD models by enhancing clearance through this key BBB transporter.

## Figures and Tables

**Table 1:** Antibody dilutions for ICC imaging of mBECs, neurons, astrocytes, and microglia.

	Primary Antibody	Secondary Alexa Fluor^®^ Antibody
**mBEC marker**	Rat anti-mouse CD31 (1:400)	Anti-rat Alexa 555 (1:500)
**Neuronal marker**	Mouse anti-human HuC/D (1:1000)	Anti-mouse Alexa 488 (1:500)
**Astrocyte marker**	Chicken anti-mouse GFAP (1:2000)	Anti-chicken Alexa 647 (1:500)
**Microglial marker**	Rabbit anti-mouse Iba1 (1:2000)	Anti-rabbit Alexa 488 (1:500)

**Table 2:** Metallomic analysis of tissue measured by ICP-MS. Concentrations of metal ions in various organs of C57BL/6J mice following a 28-day treatment with vehicle (SSV) or Cu(ATSM) (30 mg/kg/day) by oral gavage.

Tissue	Element	Concentration in Vehicle-Treated Mice (*n* = 6–8)	Concentration in Cu(ATSM)-Treated Mice (*n* = 6–8)	*p* Value	Fold Change to Control (If Significant)
**Microvessel** **Enriched** **Fraction** **(μg/mg protein)**	Sodium	237,338 ± 38,901	230,820 ± 37,462	0.9063	
	Magnesium	280.3 ± 43.39	232.3 ± 25.60	0.3634	
	Phosphorus	20521 ± 2428	19953 ± 2564	0.8754	
	Potassium	9762 ± 1065	9590 ± 1095	0.9122	
	Calcium	1922 ± 542.1	1579 ± 273.2	0.5838	
	Iron	183.8 ± 8.56	180.6 ± 10.35	0.8166	
	Copper	1.74 ± 0.20	6.16 ± 0.88	0.0006	3.5
	Zinc	13.08 ± 2.07	13.82 ± 2.01	0.8038	
**Small Intestines** **(μg/g tissue)**	Sodium	463.6 ± 34.09	401.6 ± 17.80	0.1295	
	Magnesium	86.47 ± 4.91	92.14 ± 6.67	0.5046	
	Phosphorus	1222 ± 59.09	1082 ± 78.75	0.1746	
	Potassium	1394 ± 73.55	1163 ± 84.03	0.0578	
	Calcium	8226 ± 775.9	8970 ± 1133	0.5966	
	Iron	7.558 ± 0.57	7.558 ± 0.80	0.9997	
	Copper	1.473 ± 0.10	2.545 ± 0.26	0.0016	1.7
	Zinc	8.417 ±0.40	7.796 ± 0.56	0.3817	
**Liver** **(μg/g tissue)**	Sodium	405.7 ± 13.95	398.1 ± 17.36	0.7376	
	Magnesium	87.21 ± 2.48	77.25 ± 0.70	0.0017	0.9
	Phosphorus	1310 ± 36.79	1181 ± 19.62	0.0081	0.9
	Potassium	1547 ± 43.34	1355 ± 20.16	0.0012	0.9
	Calcium	1128 ± 97.08	1363 ± 125.6	0.1619	
	Iron	56.47 ± 2.99	46.99 ± 3.00	0.0421	0.8
	Copper	3.03 ± 0.12	60.19 ± 7.89	<0.0001	19.9
	Zinc	10.94 ± 0.31	13.77 ± 0.24	<0.0001	1.3
**Kidneys** **(μg/g tissue)**	Sodium	428.2 ± 15.71	382.6 ± 30.94	0.2103	
	Magnesium	49.20 ± 2.26	41.89 ± 3.60	0.1076	
	Phosphorus	816.1 ± 36.45	702.3 ± 64.41	0.1463	
	Potassium	868.3 ± 27.34	754.0 ± 58.65	0.0991	
	Calcium	2675 ± 203.8	2306 ± 178.0	0.1946	
	Iron	22.83 ± 1.23	20.38 ± 2.48	0.3910	
	Copper	1.781 ± 0.08	1.673 ± 0.16	0.5590	
	Zinc	4.380 ± 0.18	3.777 ± 0.32	0.1238	

Data are presented as mean ± SEM, with *p* < 0.05 deemed significantly different to the vehicle-treated group assessed by an unpaired *t*-test.

## Data Availability

Data is contained within the article. The data and analysis presented in this study are available upon request.

## References

[1] Hawkins B.T., Davis T.P. (2005). The blood-brain barrier/neurovascular unit in health and disease. Pharmacol. Rev..

[2] Schinkel A.H. (1999). P-Glycoprotein, a gatekeeper in the blood–brain barrier. Adv. Drug Deliv. Rev..

[3] Ueda K., Clark D.P., Chen C.J., Roninson I.B., Gottesman M.M., Pastan I. (1987). The human multidrug resistance (mdr1) gene. cDNA cloning and transcription initiation. J. Biol. Chem..

[4] Borst P., Schinkel A.H. (2013). P-glycoprotein ABCB1: A major player in drug handling by mammals. J. Clin. Investig..

[5] Schinkel A.H., Wagenaar E., van Deemter L., A Mol C., Borst P. (1995). Absence of the mdr1a P-Glycoprotein in mice affects tissue distribution and pharmacokinetics of dexamethasone, digoxin, and cyclosporin A. J. Clin. Investig..

[6] Schinkel A., Smit J., van Tellingen O., Beijnen J., Wagenaar E., van Deemter L., Mol C., van der Valk M., Robanus-Maandag E., Riele H.T. (1994). Disruption of the mouse mdr1a P-glycoprotein gene leads to a deficiency in the blood-brain barrier and to increased sensitivity to drugs. Cell.

[7] Schinkel A.H., Wagenaar E., Mol C.A., Van Deemter L. (1996). P-glycoprotein in the blood-brain barrier of mice influences the brain penetration and pharmacological activity of many drugs. J. Clin. Investig..

[8] Callaghan R., Luk F., Bebawy M. (2014). Inhibition of the multidrug resistance P-glycoprotein: Time for a change of strategy?. Drug Metab. Dispos..

[9] Atadja P., Watanabe T., Xu H., Cohen D. (1998). PSC-833, a frontier in modulation of P-glycoprotein mediated multidrug resistance. Cancer Metastasis Rev..

[10] Palmeira A., Sousa E., Vasconcelos M.H., Pinto M.M. (2012). Three decades of P-gp inhibitors: Skimming through several generations and scaffolds. Curr. Med. Chem..

[11] Abbott N.J., Patabendige A.A.K., Dolman D.E.M., Yusof S.R., Begley D.J. (2010). Structure and function of the blood–brain barrier. Neurobiol. Dis..

[12] Choi H., Lee E.-H., Han M., An S.-H., Park J. (2019). Diminished expression of P-glycoprotein using focused ultrasound is associated with JNK-dependent signaling pathway in cerebral blood vessels. Front. Neurosci..

[13] Correia A., Monteiro A., Silva R., Moreira J., Lobo J.S., Silva A. (2022). Lipid nanoparticles strategies to modify pharmacokinetics of central nervous system targeting drugs: Crossing or circumventing the blood-brain barrier (BBB) to manage neurological disorders. Adv. Drug Deliv. Rev..

[14] Karamanos Y., Gosselet F., Dehouck M.-P., Cecchelli R. (2014). Blood–brain barrier proteomics: Towards the understanding of neurodegenerative diseases. Arch. Med. Res..

[15] Vautier S., Fernandez C. (2009). ABCB1: The role in Parkinson’s disease and pharmacokinetics of antiparkinsonian drugs. Expert Opin. Drug Metab. Toxicol..

[16] Qosa H., Lichter J., Sarlo M., Markandaiah S.S., McAvoy K., Richard J.-P., Jablonski M.R., Maragakis N.J., Pasinelli P., Trotti D. (2016). Astrocytes drive upregulation of the multidrug resistance transporter ABCB1 (P-glycoprotein) in endothelial cells of the blood–brain barrier in mutant superoxide dismutase 1-linked amyotrophic lateral sclerosis. Glia.

[17] van Assema D.M., Lubberink M., Bauer M., van der Flier W.M., Schuit R.C., Windhorst A.D., Comans E.F., Hoetjes N.J., Tolboom N., Langer O. (2011). Blood–brain barrier P-glycoprotein function in Alzheimer’s disease. Brain.

[18] Sweeney M.D., Zhao Z., Montagne A., Nelson A.R., Zlokovic B.V. (2018). Blood-brain barrier: From physiology to disease and back. Physiol. Rev..

[19] Zlokovic B.V. (2005). Neurovascular mechanisms of Alzheimer’s neurodegeneration. Trends Neurosci..

[20] Montagne A., Barnes S.R., Sweeney M.D., Halliday M.R., Sagare A.P., Zhao Z., Toga A.W., Jacobs R.E., Liu C.Y., Amezcua L. (2015). Blood-brain barrier breakdown in the aging human hippocampus. Neuron.

[21] Iturria-Medina Y.I., Sotero R.C., Toussaint P.J., Mateos-Pérez J.M., Evans A.C. (2016). Early role of vascular dysregulation on late-onset Alzheimer’s disease based on multifactorial data-driven analysis. Nat. Commun..

[22] Lam F.C., Liu R., Lu P., Shapiro A.B., Renoir J.M., Sharom F.J., Reiner P.B. (2001). β-amyloid efflux mediated by P-glycoprotein. J. Neurochem..

[23] Kuhnke D., Jedlitschky G., Grube M., Krohn M., Jucker M., Mosyagin I., Cascorbi I., Walker L.C., Kroemer H.K., Warzok R.W. (2007). MDR1-P-glycoprotein (ABCB1) mediates transport of Alzheimer’s amyloid-β peptides—Implications for the mechanisms of Aβ clearance at the blood–brain barrier. Brain Pathol..

[24] Hartz A.M., Miller D.S., Bauer B. (2010). Restoring blood-brain barrier P-glycoprotein reduces brain amyloid-β in a mouse model of Alzheimer’s disease. Mol. Pharmacol..

[25] Chai A.B., Hartz A.M.S., Gao X., Yang A., Callaghan R., Gelissen I.C. (2021). New evidence for P-gp-mediated export of amyloid-β peptides in molecular, blood-brain barrier and neuronal models. Int. J. Mol. Sci..

[26] Cirrito J.R., Deane R., Fagan A.M., Spinner M.L., Parsadanian M., Finn M.B., Jiang H., Prior J.L., Sagare A., Bales K.R. (2005). P-glycoprotein deficiency at the blood-brain barrier increases amyloid-β deposition in an Alzheimer disease mouse model. J. Clin. Investig..

[27] Vogelgesang S., Cascorbi I., Schroeder E., Pahnke J., Kroemer H.K., Siegmund W., Kunert-Keil C., Walker L.C., Warzok R.W. (2002). Deposition of Alzheimer’s β-amyloid is inversely correlated with P-glycoprotein expression in the brains of elderly non-demented humans. Pharm. Genom..

[28] Chiu C., Miller M.C., Monahan R., Osgood D.P., Stopa E.G., Silverberg G.D. (2015). P-glycoprotein expression and amyloid accumulation in human aging and Alzheimer’s disease: Preliminary observations. Neurobiol. Aging.

[29] Deo A.K., Borson S., Link J.M., Domino K., Eary J.F., Ke B., Richards T.L., Mankoff D.A., Minoshima S., O’sullivan F. (2014). Activity of P-glycoprotein, a β-amyloid transporter at the blood–brain barrier, is compromised in patients with mild Alzheimer disease. J. Nucl. Med..

[30] Jeynes B., Provias J. (2011). An investigation into the role of P-glycoprotein in Alzheimer’s disease lesion pathogenesis. Neurosci. Lett..

[31] Ayton S., Lei P., Bush A.I. (2013). Metallostasis in Alzheimer’s disease. Free Radic. Biol. Med..

[32] Lutsenko S., Bhattacharjee A., Hubbard A.L. (2010). Copper handling machinery of the brain. Metallomics.

[33] Barnham K.J., Bush A.I. (2008). Metals in Alzheimer’s and Parkinson’s diseases. Curr. Opin. Chem. Biol..

[34] Barnham K.J., Bush A.I. (2014). Biological metals and metal-targeting compounds in major neurodegenerative diseases. Chem. Soc. Rev..

[35] Agarwal P., Ayton S., Agrawal S., Dhana K., Bennett D.A., Barnes L.L., Leurgans S.E., Bush A.I., Schneider J.A. (2022). Brain copper may protect from cognitive decline and Alzheimer’s disease pathology: A community-based study. Mol. Psychiatry.

[36] McInerney M.P., Volitakis I., Bush A.I., Banks W.A., Short J.L., Nicolazzo J.A. (2018). Ionophore and biometal modulation of P-glycoprotein expression and function in human brain microvascular endothelial cells. Pharm. Res..

[37] Donnelly P.S., Caragounis A., Du T., Laughton K.M., Volitakis I., Cherny R.A., Sharples R.A., Hill A.F., Li Q.-X., Masters C.L. (2008). Selective intracellular release of copper and zinc ions from bis (thiosemicarbazonato) complexes reduces levels of Alzheimer disease amyloid-β peptide. J. Biol. Chem..

[38] Fodero-Tavoletti M.T., Villemagne V.L., Paterson B.M., White A.R., Li Q.X., Camakaris J., O’keefe G., Cappai R., Barnham K.J., Donnelly P.S. (2010). Bis (thiosemicarbazonato) Cu-64 complexes for positron emission tomography imaging of Alzheimer’s disease. J. Alzheimer’s Dis..

[39] Paterson B.M., Cullinane C., Crouch P., White A.R., Barnham K.J., Roselt P.D., Noonan W., Binns D., Hicks R.J., Donnelly P.S. (2019). Modification of biodistribution and brain uptake of copper bis(thiosemicarbazonato) complexes by the incorporation of amine and polyamine functional groups. Inorg. Chem..

[40] Pyun J., McInnes L.E., Donnelly P.S., Mawal C., Bush A.I., Short J.L., Nicolazzo J.A. (2022). Copper bis (thiosemicarbazone) complexes modulate P-glycoprotein expression and function in human brain microvascular endothelial cells. J. Neurochem..

[41] Syvänen S., Lindhe O., Palner M., Kornum B.R., Rahman O., Långström B., Knudsen G.M., Hammarlund-Udenaes M. (2009). Species differences in blood-brain barrier transport of three positron emission tomography radioligands with emphasis on P-glycoprotein transport. Drug Metab. Dispos..

[42] Deo A.K., Theil F.-P., Nicolas J.-M. (2013). Confounding parameters in preclinical assessment of blood–brain barrier permeation: An overview with emphasis on species differences and effect of disease states. Mol. Pharm..

[43] Blower P.J., Castle T.C., Cowley A.R., Dilworth J.R., Donnelly P.S., Labisbal E., Sowrey F.E., Teat S.J., Went M.J. (2003). Structural trends in copper (II) bis (thiosemicarbazone) radiopharmaceuticals. Dalton Trans..

[44] Gingras B., Suprunchuk T., Bayley C.H. (1962). The preparation of some thiosemicarbazones and their copper complexes: Part III. Can. J. Chem..

[45] Hung L.W., Villemagne V.L., Cheng L., Sherratt N.A., Ayton S., White A.R., Crouch P.J., Lim S., Leong S.L., Wilkins S. (2012). The hypoxia imaging agent Cu^II^ (atsm) is neuroprotective and improves motor and cognitive functions in multiple animal models of Parkinson’s disease. J. Exp. Med..

[46] Roberts B.R., Lim N.K., McAllum E.J., Donnelly P.S., Hare D.J., Doble P.A., Turner B.J., Price K.A., Lim S.C., Paterson B.M. (2014). Oral treatment with Cu^II^ (atsm) increases mutant SOD1 in vivo but protects motor neurons and improves the phenotype of a transgenic mouse model of amyotrophic lateral sclerosis. J. Neurosci..

[47] Soon C.P., Donnelly P.S., Turner B.J., Hung L.W., Crouch P.J., Sherratt N.A., Tan J.L., Lim N.K.H., Lam L., Bica L. (2011). Diacetylbis (*N*(4)-methylthiosemicarbazonato) copper (II) (Cu^II^(atsm)) protects against peroxynitrite-induced nitrosative damage and prolongs survival in amyotrophic lateral sclerosis mouse model. J. Biol. Chem..

[48] Pan Y., Short J.L., Newman S.A., Choy K.H., Tiwari D., Yap C., Senyschyn D., Banks W.A., Nicolazzo J.A. (2018). Cognitive benefits of lithium chloride in APP/PS1 mice are associated with enhanced brain clearance of β-amyloid. Brain Behav. Immun..

[49] Pan Y., Scanlon M.J., Owada Y., Yamamoto Y., Porter C.J.H., Nicolazzo J.A. (2015). Fatty acid-binding protein 5 facilitates the blood–brain barrier transport of docosahexaenoic acid. Mol. Pharm..

[50] McInerney M.P., Pan Y., Volitakis I., Bush A.I., Short J.L., Nicolazzo J.A. (2019). The effects of clioquinol on P-glycoprotein expression and biometal distribution in the mouse brain microvasculature. J. Pharm. Sci..

[51] Yap C., Short J.L., Nicolazzo J.A. (2020). A combination of clioquinol, zinc and copper increases the abundance and function of breast cancer resistance protein in human brain microvascular endothelial cells. J. Pharm. Sci..

[52] Xiao Z., Donnelly P.S., Zimmermann M., Wedd A.G. (2008). Transfer of copper between bis (thiosemicarbazone) ligands and intracellular copper-binding proteins. Insights into mechanisms of copper uptake and hypoxia selectivity. Inorg. Chem..

[53] Dearling J.L., Lewis J.S., Mullen G.E., Welch M.J., Blower P.J. (2002). Copper bis (thiosemicarbazone) complexes as hypoxia imaging agents: Structure-activity relationships. J. Biol. Inorg. Chem..

[54] Hilton J.B., Kysenius K., Liddell J.R., Rautengarten C., Mercer S.W., Paul B., Beckman J.S., McLean C.A., White A.R., Donnelly P.S. (2020). Disrupted copper availability in sporadic ALS: Implications for Cu^II^ (atsm) as a treatment option. BioRxiv.

[55] Hilton J.B., Mercer S.W., Lim N.K.H., Faux N.G., Buncic G., Beckman J.S., Roberts B.R., Donnelly P.S., White A.R., Crouch P.J. (2017). Cu II (atsm) improves the neurological phenotype and survival of SOD1 G93A mice and selectively increases enzymatically active SOD1 in the spinal cord. Sci. Rep..

[56] McInnes L.E., Noor A., Kysenius K., Cullinane C., Roselt P., McLean C.A., Chiu F.C.K., Powell A.K., Crouch P., White J.M. (2019). Potential diagnostic imaging of Alzheimer’s disease with copper-64 complexes that bind to amyloid-β plaques. Inorg. Chem..

[57] Price K.A., Crouch P.J., Lim S., Paterson B.M., Liddell J.R., Donnelly P.S., White A.R. (2011). Subcellular localization of a fluorescent derivative of Cu II (atsm) offers insight into the neuroprotective action of Cu II (atsm). Metallomics.

[58] Bernard-Patrzynski F., Lécuyer M.-A., Puscas I., Boukhatem I., Charabati M., Bourbonnière L., Ramassamy C., Leclair G., Prat A., Roullin V.G. (2019). Isolation of endothelial cells, pericytes and astrocytes from mouse brain. PLoS ONE.

[59] Paraiso H.C., Wang X., Kuo P.-C., Furnas D., Scofield B.A., Chang F.-L., Yen J.-H., Yu I.-C. (2020). Isolation of mouse cerebral microvasculature for molecular and single-cell analysis. Front. Cell. Neurosci..

[60] Navone S.E., Marfia G., Invernici G., Cristini S., Nava S., Balbi S., Sangiorgi S., Ciusani E., Bosutti A., Alessandri G. (2013). Isolation and expansion of human and mouse brain microvascular endothelial cells. Nat. Protoc..

[61] Lee G., Schlichter L., Bendayan M., Bendayan R. (2001). Functional expression of P-glycoprotein in rat brain microglia. J. Pharmacol. Exp. Ther..

[62] Bendayan R., Ronaldson P.T., Gingras D., Bendayan M. (2006). In situ localization of P-glycoprotein (ABCB1) in human and rat brain. J. Histochem. Cytochem..

[63] Chan G.N.Y., Hoque T., Cummins C.L., Bendayan R. (2011). Regulation of P-glycoprotein by orphan nuclear receptors in human brain microvessel endothelial cells. J. Neurochem..

[64] Ding Y., Zhong Y., Baldeshwiler A., Abner E.L., Bauer B., Hartz A.M.S. (2021). Protecting P-glycoprotein at the blood–brain barrier from degradation in an Alzheimer’s disease mouse model. Fluids Barriers CNS.

[65] Hartz A.M.S., Zhong Y., Shen A.N., Abner E.L., Bauer B. (2018). Preventing P-gp ubiquitination lowers Aβ brain levels in an Alzheimer’s disease mouse model. Front. Aging Neurosci..

[66] Nwaozuzu O.M., Sellers L.A., Barrand M.A. (2003). Signalling pathways influencing basal and H_2_O_2_-induced P-glycoprotein expression in endothelial cells derived from the blood–brain barrier. J. Neurochem..

[67] Zhou Y., Zhou J., Li P., Xie Q., Sun B., Li Y., Chen Y., Zhao K., Yang T., Zhu L. (2019). Increase in P-glycoprotein levels in the blood-brain barrier of partial portal vein ligation/chronic hyperammonemia rats is medicated by ammonia/reactive oxygen species/ERK1/2 activation: In vitro and in vivo studies. Eur. J. Pharmacol..

[68] Shao Y., Wang C., Hong Z., Chen Y. (2016). Inhibition of p38 mitogen-activated protein kinase signaling reduces multidrug transporter activity and anti-epileptic drug resistance in refractory epileptic rats. J. Neurochem..

[69] Srivastava S., Blower P.J., Aubdool A.A., Hider R.C., Mann G.E., Siow R.C. (2016). Cardioprotective effects of Cu (II) ATSM in human vascular smooth muscle cells and cardiomyocytes mediated by Nrf2 and DJ-1. Sci. Rep..

[70] Acevedo K.M., Hayne D.J., McInnes L.E., Noor A., Duncan C., Moujalled D., Volitakis I., Rigopoulos A., Barnham K.J., Villemagne V.L. (2018). Effect of structural modifications to glyoxal-bis (thiosemicarbazonato) copper (II) complexes on cellular copper uptake, copper-mediated ATP7A trafficking, and P-glycoprotein mediated efflux. J. Med. Chem..

[71] Crouch P.J., Hung L.W., Adlard P.A., Cortes M., Lal V., Filiz G., Perez K.A., Nurjono M., Caragounis A., Du T. (2009). Increasing Cu bioavailability inhibits Aβ oligomers and tau phosphorylation. Proc. Natl. Acad. Sci. USA.

[72] Lin C.C., Hsieh H.L., Shih R.H., Chi P.L., Cheng S.E., Yang C.M. (2013). Up-regulation of COX-2/PGE 2 by endothelin-1 via MAPK-dependent NF-κB pathway in mouse brain microvascular endothelial cells. Cell Commun. Signal..

[73] Qin L.-H., Huang W., Mo X.-A., Chen Y.-L., Wu X.-H. (2015). LPS induces occludin dysregulation in cerebral microvascular endothelial cells via MAPK signaling and augmenting MMP-2 levels. Oxidative Med. Cell. Longev..

[74] Wang X., Campos C.R., Peart J.C., Smith L.K., Boni J.L., Cannon R.E., Miller D.S. (2014). Nrf2 upregulates ATP binding cassette transporter expression and activity at the blood–brain and blood–spinal cord barriers. J. Neurosci..

[75] Chai A.B., Callaghan R., Gelissen I.C. (2022). Regulation of P-Glycoprotein in the brain. Int. J. Mol. Sci..

[76] Zhao J., Moore A.N., Redell J.B., Dash P.K. (2007). Enhancing expression of Nrf2-driven genes protects the blood–brain barrier after brain injury. J. Neurosci..

[77] Huang H.-C., Nguyen T., Pickett C.B. (2002). Phosphorylation of Nrf2 at Ser-40 by protein kinase C regulates antioxidant response element-mediated transcription. J. Biol. Chem..

[78] Pan Y., Kagawa Y., Sun J., Turner B.J., Huang C., Shah A.D., Schittenhelm R.B., Nicolazzo J.A. (2022). Altered blood-brain barrier dynamics in the C9orf72 hexanucleotide repeat expansion mouse model of amyotrophic lateral sclerosis. Pharmaceutics.

[79] Dagenais C., Rousselle C., Pollack G.M., Scherrmann J.M. (2000). Development of an in situ mouse brain perfusion model and its application to mdr1a P-glycoprotein-deficient mice. J. Cereb. Blood Flow Metab..

[80] Cherny R.A., Atwood C.S., E Xilinas M., Gray D.N., Jones W.D., A McLean C., Barnham K.J., Volitakis I., Fraser F.W., Kim Y.-S. (2001). Treatment with a copper-zinc chelator markedly and rapidly inhibits β-amyloid accumulation in Alzheimer’s disease transgenic mice. Neuron.

[81] Elmeliegy M., Vourvahis M., Guo C., Wang D.D. (2020). Effect of P-glycoprotein (P-gp) inducers on exposure of P-gp substrates: Review of clinical drug–drug interaction studies. Clin. Pharmacokinet..

[82] Stamoulis I., Kouraklis G., Theocharis S. (2007). Zinc and the liver: An active interaction. Dig. Dis. Sci..

[83] Hatano R., Ebara M., Fukuda H., Yoshikawa M., Sugiura N., Kondo F., Yukawa M., Saisho H. (2000). Accumulation of copper in the liver and hepatic injury in chronic hepatitis C. J. Gastroenterol. Hepatol..

[84] Nikseresht S., Hilton J.B., Liddell J.R., Kysenius K., Bush A.I., Ayton S., Koay H., Donnelly P.S., Crouch P.J. (2023). Transdermal application of soluble Cu^II^ (atsm) increases brain and spinal cord uptake compared to gavage with an insoluble suspension. Neuroscience.

